# Flexible Integration of Laser Myringotomy and Ventilation Tube for Bilateral Otitis Media with Effusion: Analysis of Laser Tympanostomy versus Ventilation Tube

**DOI:** 10.1371/journal.pone.0084966

**Published:** 2014-01-23

**Authors:** Chang Ho Lee, Jun Ho Lee, Hyoung-Mi Kim

**Affiliations:** 1 Department of Otolaryngology, CHA Bundang Medical Center, CHA University, Seongnam-si, Republic of Korea; 2 Department of Pediatrics, CHA Bundang Medical Center, CHA University, Seongnam-si, Republic of Korea; Harvard Medical School, United States of America

## Abstract

**Background:**

The aim of this study was to evaluate the efficacy of laser myringotomy (LM) compared to ventilation tube (VT), and to assess the clinical success criteria of LM-assisted VT insertion as the flexible alternatives avoiding GA for the treatment of bilateral consistent otitis media with effusion (OME).

**Methods and Findings:**

LM under topical anesthesia was followed by VT insertion in cooperative children using Acuspot® 712 CO2 laser micromanipulator attached microscope. Sixty children failed VT and bilateral laser tympanostomy was done (group LL), and 130 children tolerated VT on one side but LM on the other side (group LV). The efficacy of LM was compared to VT regarding recurrent effusion and reoperation as the outcome measure**;** firstly, by ear-to-ear matched pair analysis in LV, and secondly between LL vs. LV. Long-term outcome was compared to control group who received both VT under GA (group GAVT) regarding the outcome of additional VT and GA.

**Results:**

The effectiveness of LM per ear was equivocal as 46.9% and 40.8% in LV and LL respectively; but the effectiveness per children was further lower in LL as 28.3%, which was a limitation of LM for bilateral OME. LL required reoperation in 71.7% mostly requiring impending GA in 51.7% within 4.7 months, thus was a controversial treatment. But LV required GA in 20.8% during the 27.2 months long-term follow-up, which was second set of VT and adenoidectomy that were also comparably required in GAVT control with multiple GA.

**Conclusion:**

Standard GAVT was more recommended for bilateral OME than bilateral LM (LL) in our practice. But LM was selectively effective for some children, that combined approach with LM plus VT provided comparable period to outgrow OME as effectively as GAVT, when LM was supplemented with one VT side with recovered hearing.

## Introduction

Otitis media with effusion (OME) is the most common cause of hearing loss and ventilation tubes (VT) is accepted as a standard surgical treatment, which is the most common operation in young children requiring multiple general anesthesia (GA) [1] [2]. Laser myringotomy (LM) was introduced as an option for office-based ventilation under topical anaesthesia (TA) to avoid the concerns of families who refuse VT and GA [3]. The efficacy of LM without VT was reported to be 40% resulting in frequent failures, which is roughly between that of knife myringotomy (KM) and VT insertion [4]. LM-assisted VT insertion overcame the frequent LM failure and reduced GA, but the limited feasibility of LM-assisted VT in young children requires the integration of two procedures [5]. In our clinic, these children have been allocated to “flexible integration of laser tympanostomy and ventilation tube insertion under topical anaesthesia” (FITT) procedure before definitive GA procedure [6]. We have reported that LM-assisted VT insertion was feasible in 73% for one VT, and 45% for both VT, which could reduce 80% of required GA in prospective studies [7] [8]. The role of LM in this integrated approach is to facilitate VT insertion when feasible, or to provide short-term ventilation if VT is not feasible. FITT is a fitting procedure before definite general anesthesia, and the aim of FITT is 1) To stop antibiotic overuse 2) To restore normal hearing promptly with VT insertion 3) To assess the outcome of VT: duration until extrusion, otorrhea 4) To decide the need for long-term treatment such as adenoidectomy or long-term tube.

The aim of this study was to evaluate the efficacy of LM compared to ventilation tube (VT), and to assess the clinical success criteria of LM-assisted VT insertion as the flexible alternatives avoiding GA for the treatment of bilateral consistent otitis media with effusion (OME).

## Methods

Our surgical procedure and protocol of FITT was published in another study [6]. Feasibility criteria of FITT was 1) the child could tolerate an otomicroscopic exam and 2) the child’s ear canal permitted entry of a 5 mm-diameter Hartman round speculum (#2, Storz Item #N0241-2) or 4 mm otoendoscope. A Sharplan CO_2_ laser (Model 30C, Allendale, U.S.A.), in conjunction with Acuspot® 712 micromanipulator attached to a Zeiss microscope (ProErgo®/S7, LLC, USA) was used with the following settings: 10–20W power; 0.10–0.15 seconds in duration; and a single, defocused pulse. The size of the LM was made smaller than the outer flange size of Paparella type I VT (2.5 mm) to prevent VT extrusion. In addition to LM-only procedures, the effusion was aspirated with an 18 G suction needle to allow easy insertion of the VT.

Study inclusion criteria included the following: children younger than 7 years, persistent bilateral OME >4 months duration, with bilateral B tympanometry, HL ≥25 dB documented by PTA, play audiometry, or OAE; ≥12 months of follow-up; and attendance until the end of follow-up. Study exclusion criteria included the following: unilateral OME, recurrent OME; previous adenoidectomy or VT, bifid uvula, cleft palate. Treatment failure was defined as reoperation or recurrent effusion with ≥25 dB HL. The end of follow-up was until 24 months if reoperation was not done, or until the time second VT procedure if reoperation was performed.

Table 1 summarized the method of integrating LM and VT flexibly according to the tolerance of each child. A review of FITT surgical database from 2004 to 2010 found 60 children treated with bilateral laser tympanostomy without VT (group LL), 130 children with LT/VT (group LV), and 332 children with bilateral VT (not analyzed in this study) during the same period. The treatment outcome of group LL and LV was compared to control group of 50 children who received VT under GA (GAVT).

**Table 1:** Performed procedure in “flexible integration of laser tympanostomy and tube” (FITT) that was variable by the degree of feasibility under topical anesthesia.

Study group inthis paper	Performedprocedure	Childtolerance	Feasibilityof VT	Percentage in ourpractice^a^
Not analyzed	VT/VT	Good	Success in both VT	52.9%
LV	LT/VT	Fair	VT in one ear, Fail VT in the other ear	23.5%
LL	LT/LT	Poor	Failure in both VT	11.7%
GAVT Control	VT under generalanesthesia	No	Failure to start procedure undertopical anesthesia	11.7%

VT = ventilation tube insertion after laser myringotomy, LT = laser tympanostomy without VT.

^a^ From a preliminary study of children 36∼60 months old [7].

### Ethical Statement

VT under GA requires blood sampling, maintenance of an intravenous line, two-day admission to a hospital, fasting from midnight until surgery, and endotracheal intubation. Thus, FITT was considered definitely less invasive. The Internal Review Board of CHA University approved this study (CHA IRB No. BD2010-085D), and written informed consent was obtained from the children’s legal guardians.

### Statistical Analysis

SAS Version 9.1 (SAS Institute Inc., Cary, NC) was used for statistical analysis. Kruskal – Wallis and multiple Mann-Whitney test were used for comparison of differences in age, disease duration and hearing threshold among groups. Paired t-test was used to compare the response to treatment of each ear in the same individual. Chi-square test was applied to analyze treatment outcomes among groups including LL, LV, GAVT and controls. P values <0.05 were deemed statistically significant.

## Results

Table 2 summarized preoperative profile of study group LL, LV, and control group GAVT. Each group consisted of consecutive surgical cases that did not differ in the disease severity, and differed only in the feasibility as described in Table 1. LV group was older than control group LL or GAVT; because in our FITT 1) VT was seldom feasible for children younger than 12 months 2) GAVT was not frequently performed for children older than 4 years. Preoperative severity of OME assessed by past duration of effusion and hearing threshold was not different between each group. All ears in LL and LV had positively confirmed B type tympanometry and more glue-like effusion than GAVT control, which was the advantage of FITT without time delay between diagnosis and surgery. The efficacy of LM was compared to VT in two-way; 1) matched pair ear-to-ear analysis in LV, 2) group LL vs. LV.

**Table 2:** Patient demographics and preoperative profile of study group “Laser myringotomy plus ventilation tube” (LV) vs. “Bilateral Laser myringotomy” (LL) compared to control group “ventilation tube under general anesthesia” (GAVT).

	LL	LV	GAVT	*p*
**N**	60 children	130 children	50 children	
Age (months)	36.3±19.4	51.1±15.9*	39.5±18.6	*p<0.005
Age range (months)	6∼83	15∼82	9∼83	
0∼12	*9*	0	2	
13∼24	*6*	11	11	
25∼36	*13*	9	6	
37∼48	*10*	33	18	
49∼60	*6*	36	6	
61∼83	*16*	41	7	
Past OME duration (months, range)	9.1±6.1 (4∼24)	7.4±5.2 (4∼24)	8.1±8.6 (4∼26)	*NS*
Hearing threshold	In 12 children	In 55 children	In 14 children	
Worse ear	*29.0*±*5.8*	34.1±8.2	34.3±6.3	*NS*
Better ear	*22.8*±4.2	24.5±8.3	28.2±7.3	*NS*
B type tympanometry	All	All	82	
Glue-like effusion (ears)	72	162	65	

NS = not significantly different.

The efficacy and recurrence interval of LM per ear was obtained in comparison to VT extrusion by ear-to-ear matched pair analysis in group LV (Table 3). Recurrent OME on LM was readily observed in 36.9% within 2 months, and increased to 62.3%. This efficacy of LM was significantly lower than VT extrusion until 6 months, even though VT extrusion was not always VT failure. Early closure of LM in 2.4 weeks required significantly more reoperation, but significantly less complication. There was also 24.6% early extrusion of VT before 6 months that could also be regarded as a complication of VT. LM side did not develop residual perforation or any major complications. The effectiveness of LM vs. VT per ear was 46.9% vs. 78.5% respectively, if recurrence was assessed by reoperation for >6 months follow-up.

**Table 3:** Laser myringotomy versus Ventilation tube: Matched pair analysis of ear-to-ear in the same child with bilateral positive effusion confirmed with myringotomy.

	Ear treated with Lasertympanostomy	Ear treated withVentilation tube	p
N	130 ears	130 ears	
Average duration of ventilation	2.4 weeks	9.7 months	
**Recurrent effusion vs. VT extrusion**			
0∼2 months	48 (36.9%)	12 (9.2%)	0.000
2∼4 months	60 (46.2%)	18 (13.8%)	0.000
4∼6 months	70 (53.8%)	32 (24.6%)	0.000
12 months	81 (62.3%)	92 (70.8%)	NS
**Efficacy at 6 months** (100% - recurrence)	46.2%	75.4%	0.000
**Reoperation at end of follow-up**	69(53.1%)	28 (21.5%)	0.0001
**Effectiveness at end of follow-up** (100% - Reoperation)	46.9%	78.5%	0.0001
**Major Complication**	1 (1.8%)	11 (8.5%)	0.04
Otorrhea controlled with intravenous antibiotics	0	6 (4.6%)	
Perforation	1 (1.8%)	4 (3.1%)	
Cholesteatoma	0	1 (0.8%)	

AOM = acute otitis media, NS = not significant.

Table 4 compared the treatment outcome of group LL vs. LV, which compared the efficacy of bilateral LM to unilateral LM per children. The rate of recurrent effusion on LM side was equivocal between group LL vs. LV, but WW for recurrent OME (rOME) significantly increased in group LV because parents selected WW more frequently when the child had VT (and thus normal hearing), and GAVT was not readily preferred. Group LV required reoperation significantly less in 53.1% (p = 0.02) and ultimately GA was also significantly reduced to 20.8% even though the child had only one VT (p = 0.003), that was clinically acceptable. Compared to LV, children treated with LM only (group LL) usually required reoperation in 76.7% and GA in 51.7%, that LL was clinically regarded as a vulnerable treatment because the recurrences and the interval until GA was very brief as 4.7 months. Even in children in group LV who eventually required GA such as for second VT insertion or adenoidectomy, the interval until GA was 14.4 months, significantly longer than group LL, and watchful waiting (WW) in this interval also reduced the rate of GA.

**Table 4:** Treatment outcome of Laser myringotomy in group LL (Bilateral laser myringotomy) compared to group LV (One laser myringotomy plus one ventilation tube).

	LL	LV	*p*
N	60 children	130 children	
Follow-up duration (months, range)	15.4±15.7 (6∼60)	27.2±15.8 (12∼75)	
**Treatment outcome of LM per children**			
No recurrence	10 (16.7%)	29 (22.3%)	NS
WW for rOME	7 (11.7%)	32 (24.6%)	p = 0.04
No. of reoperation	43 (71.7%)	69 (53.1%)	p = 0.02
Reoperation VT under TA(facilitated by LM)	15 (25.0%)	42 (32.3%)	NS
Reoperation VT under GA	28 (51.7%)	27 (20.8%)	p = 0.0003
Interval until GA (months)	4.7	14.4	
**Age**	**Success of LM per children/n (%)**	**Success of LM per children/n (%)**	
0∼12 months	4/9 (44%)	0/0	
13∼24	3/6 (50%)	6/11 (54.5%)	
25∼36	3/13 (33%)	6/9 (66.7%)	
37∼48	2/10 (20%)	19/33 (57.6%)	
49∼60	1/6 (16.7%)	13/36 (36.1%)	
61∼73	4/16 (25.0%)	17/41 (41.5%)	
Total	17/60 (28.3%)	61/130 (46.9%)	p = 0.01

rOME = recurrent otitis media with effusion.

We analyzed the effectiveness of LM in each age group regarding “No recurrence” or “WW for rOME” as success, and reoperation as failure of LM. The effectiveness of LM per children was very variable (16.7%∼50.0%), and was higher in the younger (<24 months) age when bilateral LM was done in group LL. But the effectiveness of bilateral LM in group LL was lower than unilateral LM in LV (p = 0.01) in every age, meaning the effectiveness of LM was dependent on the status of the opposite ear (with VT or without VT). In group LL, the effectiveness of LM per ears was 40.8% (49/120) because 15 children required unilateral reoperation, but the effectiveness per children was 28.3%, which was significantly lower than group LV.

Table 5 summarized long-term follow-up result of LV compared to GAVT control. Second set of VT meant bilateral VT insertion after first VT extrusion, which was usually adenoidectomy under GA for group LV. The rate of second VT surgery was 26.9% in group LV not significantly different from GAVT control, probably because it was 14.4 months after first FITT procedure because children maintained good hearing as long as one VT was retained. The number of adenoidectomy was significantly lower in the LV compared to GAVT, which shows tendency to more adenoidectomy under GA to prevent multiple GA. GA was required in 20.8% for group LV, which was equivalent to children requiring multiple GA in GAVT group. Multiple GA was not necessary, while GAVT required multiple GA in 16%. The rate of complications was lower in group LV than group GAVT owing to low complication rate of LM side (Table 3).

**Table 5:** Long –term treatment outcome of “One Laser tympanostomy plus One ventilation tube” (group LV) compared to children who received bilateral ventilation tube under general anesthesia (GAVT).

Study group	LV	GAVT	*p*
n	130 children	50 children	
Follow-up duration (months, range)	27.2±15.8 (12∼75)	29.7±18.8 (12∼72)	
**Surgery performed**			
No. of 2^nd^ set of VT	35 (26.9%)	16 (32%)	NS
2^nd^ VT under GA	27	4	
2^nd^ VT under TA(facilitated by LM)	8	12	
Interval between 1^st^∼2^nd^ VT	14.4	14.3	NS
No. of 3 or more sets of VT	9 (7.0%)	4 (8%)	NS
No. of adenoidectomy	25 (19.2%)	23 (46%)	*p = 0.005*
No. of tonsillectomy	16(12.3%)	13 (26%)	p = 0.02
No. of long-term tube	12 (9.2%)	8 (16%)	*NS*
**GA**			
GA actually performed			
GA once	27	All	
Multiple GA	none	8 (16%)	
Total No. of GA	27 (20.8%)	58 (116%)	
**Complication**	11 (8.5%)	7 (14%)	NS
Otorrhea	6	2	
Perforation	4	4	
Cholestaetoma	1	1	

VT = Ventilation tube, GA = general anesthesia, TA = topical anesthesia, NS = not significant.

## Discussion

This study is the first matched pair study on the efficacy of LM under topical anesthesia (TA), and there was only one such matched-pair study under GA [4]. The efficacy of LM in the published articles was summarized in Table 6, and the efficacy was largely variable in each study [4] [5] [9]–[12]. This kind of large variability seems to be channeling type of selection bias, in which favorable children were assigned to LM. Strict criteria on surgical candidates would minimize this selection bias, and this study included only children with bilateral surgically positive effusions with ≥25 dB HL of >4 months duration to exclude marginal surgical candidates. They are primary surgical candidates on current clinical practice because natural resolution during WW is least likely and would benefit most from VT, which were children younger than 3 years attending day-care, or older than 4 years with HL >25 dB for at least 12 weeks [13]. The efficacy of bilateral LM without VT (group LL) was not clinically distinguishable from spontaneous resolution achievable with WW, which was reported to be successful in 20–56% of cases without any treatment [14]–[16].

**Table 6:** Published efficacy of Laser myringotomy.

Study	Follow-up duration (mo.)	Efficacy (Success)	Recurrence Rate (Failure)	Comment
Koopman 2004 [4]	6	40%		compared to VT (having 78% efficacy)
Cotter 2004 [5]	3	42.6%46.4% in rAOM36.8% in cOME	57.4% needed VT	per child
Prokopakis 2002 [9]		51.5%		
Silverstein 2001 [11]	1	46%	49% needed VT	
Sedlmaier 2002 [10]	6		26.3% mucoid 13.5% serous	with Adenoidectomy
Cook 2001 [12]	3	83%	-	with Adenoidectomy

It is also a well-known principle that myringotomy alone is not a recommended treatment to replace the role of VT for OME, thus we preferred to insert VT if possible rather than leave LM close itself shortly in 2 weeks. We hypothesized that LM was better to be accompanied by VT insertion if feasible because LM was not so effective but useful for supplementing difficult VT procedure under TA. Matched analysis comparison of LM vs. VT in group LV (Table 4) showed the addition of VT could complement early failure cases of LM. The efficacy of LM per ear from matched pair study including our data in Table 3 seems to be more accurate, but it also has a limitation because the intervention of the study is unilateral, that we compared the outcome of various treatment groups to assess the effectiveness per children. We also hypothesized FITT might enable intelligent decision on early intervention with adenoidectomy or long-term VT placement without multiple GA in the long-term. Group LV showed decreased adenoidectomy and multiple GA compared to standard GAVT control (Table 5).

To insert VT without GA is challenging but the demand seems to be increasing because; 1) VTs are frequently repeated surgery in the children at risk with OME 2) Meta-analysis or long-term outcome studies favor WW rather than VT in otherwise healthy children, and then these children also do not have to take the risk of GAVT [17]. The feasibility of KM and VT insertion was 95% in older children (>8 yrs) and adults under TA, but KM is not safe enough in younger children under TA [18]. VT is repeated under GA in 20∼50% of suffering subgroup of children requiring additional bout of GA and VT [19], and a large cohorts study suggested multiple GA in young children might be related to behavioral problem such as attention-deficit hyperactivity disorder [20] [21]. The advent of LM has renewed interest in VT insertion under TA [22], and 70% of younger children did not report severe pain with LM assisted VT [3]. LM-assisted VT is minimally invasive and parental satisfaction has been consistently reported if it is feasible [3] [5] [23]. LM-assisted VT insertions has additional advantage of less intraoperative bleeding and lower postoperative otorrhea compared to KM [24].

Bilateral VT under TA would be desirable and equally effective as GAVT, but our previous study showed that it was tolerable in only half of the children. However we found in this study that unilateral VT in group LV provided period comparable to GAVT in the long-term follow-up. LV frequently required re-operation for LMs that recurred, but immediate GA was not necessary because recurrence on LMs were assigned to WW as if unilateral OME was with normal hearing obtained on the VT side. As a result, 79.2% of LV actually did not require procedure under GA over 2-year follow-up while 51.7% of bilateral LM (group LL) required impending GA in 4.7 months. Some concern for group LV might be increased complication on the LM side, but our results in Table 5 showed that LV was also equally effective as managing OME regarding the rate of second VT, moreover with less complication. Recent systemic review suggested that VT is also controversial, because after six to nine months by which time natural resolution also leads to improved hearing in the non-surgically treated children [17]. There was also a 10-fold difference in the rates of VT recommendation, which implies difficulty and uncertainty of clinical decision about who benefits most from VT under GA [25]. Even longer period of WW is recommended for non-severe subgroup of OME concerning the overuse of VT [19] [26]. Reduced GA itself is of course minimally invasive without intravenous catheter and endotracheal intubation [27], but FITT also significantly reduced the rate of adenoidectomy because adenoidectomy was strictly preserved until second VT. But the complication should be evaluated per individual, and GAVT is a standard treatment for children at risk to develop complications.

CO2 laser assisted VT insertion requires expensive surgical microscope and laser adaptor, but the treatment is not an expensive procedure if these equipment are commonly utilized for laryngeal or otosclerosis surgery. Under TA, VT insertion is not always possible, and then LM is also used flexibly even though not intended, thus I dubbed the term FITT for the topical procedures. FITT has two advantages, firstly to insert VT without GA, and secondly to assess the severity of OME as to require VT when LM was done instead of VT. FITT does not aim to avoid VT insertion, and in this respect FITT is different from OtoLAM® or LM only treatment, which was frequently cited to avoid VT insertion. When VT was not feasible without GA, LM was an ad hoc procedure to see if the child recurs with persistent effusion after LM has cleared the effusion and edematous middle ear mucosa and eustachian tube. It is not a generalization that LM was an effective treatment in every situation because LM only treatment such as group LL in this study did not show any advantage over spontaneous resolution or WW. But I think that there might be some LM-responsive group even in bilateral OME indicated for VT according to current surgical guidelines, that LM might find its utility. Surgical treatment of OME is like a bargaining with the parents, and LM is one such tool because the decision is also influenced by parent side satisfactions, thus the efficacy of LM seems to be dependent on how much the parents are satisfied to consent to WW or continued surgical treatment.

## Conclusion

Considering the low efficacy of bilateral LM resulting in frequent recurrence and early reoperation, standard GAVT or WW was more recommended for bilateral OME than laser tympanostomy alone (LL) in our practice. But LM was selectively effective for some children, that combined approach with LM plus VT provided comparable period to outgrow OME as effectively as GAVT, when LM was supplemented with one VT side with recovered hearing. FITT was worth attempting to reduce the multiple GA required for repeated VT insertion in high-risk children. FITT was less invasive than GAVT for non-severe subgroup of OME because extended WW could decrease the number of GA or adenoidectomy.
